# How ambient temperature affects the heading date of foxtail millet (*Setaria italica*)

**DOI:** 10.3389/fpls.2023.1147756

**Published:** 2023-03-02

**Authors:** Ya-Chen Huang, Yu-tang Wang, Yee-ching Choong, Hsin-ya Huang, Yu-ru Chen, Tzung-Fu Hsieh, Yann-rong Lin

**Affiliations:** ^1^ Department of Agronomy, National Taiwan University, Taipei, Taiwan; ^2^ Crop Science Division, Taiwan Agricultural Research Institute, Taichung, Taiwan; ^3^ Department of Plant and Microbial Biology, North Carolina State University, Raleigh, NC, United States; ^4^ Plants for Human Health Institute, North Carolina State University, Kannapolis, NC, United States; ^5^ Headquarters, World Vegetable Center, Tainan, Taiwan

**Keywords:** ambient temperature, foxtail millet, gene × environment interaction, gene × gene interaction, heading date

## Abstract

Foxtail millet (*Setaria italica*), a short-day plant, is one of the important crops for food security encountering climate change, particularly in regions where it is a staple food. Under the short-day condition in Taiwan, the heading dates (HDs) of foxtail millet accessions varied by genotypes and ambient temperature (AT). The allelic polymorphisms in flowering time (FT)–related genes were associated with HD variations. AT, in the range of 13°C–30°C that was based on field studies at three different latitudes in Taiwan and observations in the phytotron at four different AT regimes, was positively correlated with growth rate, and high AT promoted HD. To elucidate the molecular mechanism of foxtail millet HD, the expression of 14 key FT-related genes in four accessions at different ATs was assessed. We found that the expression levels of *SiPRR95*, *SiPRR1*, *SiPRR59*, *SiGhd7-2*, *SiPHYB*, and *SiGhd7* were negatively correlated with AT, whereas the expression levels of *SiEhd1*, *SiFT11*, and *SiCO4* were positively correlated with AT. Furthermore, the expression levels of *SiGhd7-2*, *SiEhd1*, *SiFT*, and *SiFT11* were significantly associated with HD. A coexpression regulatory network was identified that shown genes involved in the circadian clock, light and temperature signaling, and regulation of flowering, but not those involved in photoperiod pathway, interacted and were influenced by AT. The results reveal how gene × temperature and gene × gene interactions affect the HD in foxtail millet and could serve as a foundation for breeding foxtail millet cultivars for shift production to increase yield in response to global warming.

## 1 Introduction

Foxtail millet [*Setaria italica* (L.) P. Beauv.] is the second most cultivated millet after pearl millet, widely cultivated in China, India, Indonesia, the Korean peninsula, and southern Europe (FAOSTAT; http://faostat.fao/org/. Foxtail millet is a nutrient-rich food source that is high in essential amino acids, vitamins, and minerals compared with most cereal grains (Sachdev et al., 2021). Foxtail millet grains have become a good option for healthy diets because of its gluten-free protein, low glycemic index (GI), and high fiber content (Lestari et al., 2017; Yin et al., 2019).

Foxtail millet was domesticated from its wild ancestor, green foxtail [*S. viridis* (L.) P. Beauv.], which is evolutionarily close to several cereals and energy crops and considered as the model C4 plants (Doust et al., 2009; Jia et al., 2013). Numerous foxtail millet accessions have been collected worldwide and preserved as valuable germplasms, and phenotypic and genotypic variations also indicate a very high genetic diversity (Li and Wu, 1996; Reddy et al., 2006; Doust et al., 2009). In comparison with maize, wheat, and sorghum, foxtail millet has high water usage efficiency, which is recognized as a drought-tolerant crop (Lata et al., 2013; Kumar et al., 2018). Because of high adaptability in diverse and harsh environments, foxtail millet as a staple food has been brought to attention to cope with climate changes for food security (Lata et al., 2013; Kumar et al., 2018). These agronomic and nutritional characteristics of foxtail millet are of great significance in the agricultural economy (Bandyopadhyay et al., 2017).

Flowering time (FT) is one major characteristic for plants adaptation in wide-range geographic distribution and is also an important trait for crop management and production. FT is a complex trait regulated by numerous genes, the environment, and gene–environment interactions. Many genetic mapping and molecular physiology studies have been performed in crops and exemplified in the model plants Arabidopsis and rice. In Arabidopsis, which required vernalization and long day (LD) for flowering, multiple genetic pathways regulate the response to internal or external cues, including photoperiod, temperature, vernalization, autonomous, and hormone pathways (Araki, 2001). Compared with the regulation of flowering in Arabidopsis under LD conditions, studies on flowering in rice and temperate cereals under short-day (SD) conditions also highlight the conservation of their regulation (Cao et al., 2021). Integrating with two major genes, *FLOWERING LOCUS T* (*FT*) and *SUPPRESSOR OF OVEREXPRESSION OF CO1* (*SOC1*), regulates the transition from the vegetative growth stage to the reproductive stage (Cho et al., 2017). The *CO*-*FT* system is conserved among angiosperms. Rice, an SD monocot model plant, has a *Heading date 1 (OsHd1)–Heading date 3a (OsHd3a)* pathway, which is homologous to the *AtCO-AtFT* pathway in Arabidopsis (Cao et al., 2021). However, rice has another unique pathway, the *grain number*, *plant height*, *and heading date 7 (OsGhd7)–Early heading date 1 (OsEhd1)–OsHd3a* pathway (Itoh et al., 2010; Zhao et al., 2015). The FT pathway research in foxtail millet is still limited. Several FT quantitative trait loci (QTLs) regions were first identified by a cross between foxtail millet and its wild relative green foxtail, including some candidate genes from autonomous and photoperiod pathway such as *AtCO* and *AtFT/OsHD3A/OsRFT1* (Mauro-Herrera et al., 2013). The genome-wide association studies (GWAS) on foxtail millet revealed candidate genes underlying FT such as pseudo-response regulator gene, *SiPRR37* (Jia et al., 2013; Mauro-Herrera et al., 2013; Jaiswal et al., 2019; Li et al., 2021; Fukunaga et al., 2022).

Studies of FT-related traits in foxtail millet landraces collected from various regions in Europe and Asia revealed that the variations in HD, floral initiation, and panicle development are influenced by temperature and daylength (Takei and Sakamoto, 1987; Takei and Sakamoto, 1989). Ambient temperature (AT) and photoperiod are the two critical environment cues that affect FT, and AT has recently attracted much attention due to global climate change. Raising AT also promotes flowering in Arabidopsis and rice (Balasubramanian et al., 2006; Shah et al., 2014). The AT increased in Northern Europe from 1985 to 2009, causing oats, wheat, and maize to heading approximately 1–3 weeks earlier and yields decreased (Olesen et al., 2012; Jagadish et al., 2016). Several genes regulated by AT were identified (Thines et al., 2014; Jung et al., 2016; Legris et al., 2016; Qiu et al., 2019; Nagalla et al., 2021). *PHYTOCHROME B (AtPHYB)*, a photoreceptor, is known to act as a temperature sensor at night (Jung et al., 2016; Legris et al., 2016) and senses daytime temperature through *PHYTOCHROME INTERACTING FACTOR4 (AtPIF4)*, thereby enhancing *AtFT* expression (Thines et al., 2014; Qiu et al., 2019). *OsPHYB* as an AT sensor increases *OsGhd7* repressor activity at low AT to delay FT but decreases *OsGhd7* at a high AT to promote FT (Xue et al., 2008; Nagalla et al., 2021).

FT is regulated by the coordination of local environmental signal and plant genetic information. In addition, their genotype-by-environment (G × E) interactions are crucial for local adaptation. The genetic diversity of Taiwan foxtail millet is very high due to adaptation to various environment and selection by various preferences of indigenous peoples (Kuo et al., 2018). In our previous study, HDs varied widely among 133 Taiwan foxtail millet accessions, ranging from 53.7 to 109.5 days (Chen et al., 2017). Foxtail millet, an SD plant, is cultivated in Taiwan under the SD condition with the day length in the range of 10.5–11.5 h. The HD variations could be accounted by different genotypes and AT. The major factors affecting HD variation are genetic diversity and AT, not photoperiod. Thus, this study aimed to understand how genotypes of foxtail millet, AT, and their G × E effects lead to HD variation. Understanding the G × E effects on HD or FT is important for improving the crops under the future climate change conditions.

## 2 Materials and methods

### 2.1 Plant materials and growth environments

The foxtail millet accessions including five landraces collected from the eastern, southern, and central Taiwan (Arie, Tabungieth, Ch’I pa ran, Masunglab, and WF0677), two Taiwan cultivars (TT8 and TCS1), and two landraces from India (Bihar and Andhra Pradesh) were requested from the National Plant Genetic Resources Center, Taiwan Agricultural Research Institute. These nine accessions were cultivated in three locations: Taiwan Agricultural Research Institute, Taichung (TC; 24°N 120°E); Chiayi Agricultural Experiment Branch, Chiayi (CY; 23°N 120°E); and National Taiwan University Experimental Farm, Taipei (TP; 25°N 121°32E).

The meteorological data were retrieved from the environmental resource database of the Environmental Protection Administration (https://erdb.epa.gov.tw), including hourly temperature, relative humidity, precipitation, and sunshine duration. All the meteorological data from foxtail millet cultivated in each cultivation area for the first 2 months, collected from the nearest stations of the cultivation fields, such as the data from the TC station on 11 November 2016 to 11 January 2017, the CY station on 2 October 2018 to 2 December 2018, and the TP station on 6 January 2020 to 6 March 2020.

Four accessions exhibiting diverse HDs were selected for studying the effect of temperature on plant growth and HD. These four accessions—Taiwan landrace Arie, India landrace Andhra Pradesh, and two Taiwan cultivars (TT8 and TCS1)—were cultivated under five temperature regimes in the phytotron of National Taiwan University from 11 February to 31 August 2022. The environment conditions in the phytotron were under natural daylight with controlled day/night temperatures of 35/30°C, 30/25°C, 25/20°C, 20/15°C, and 15/13°C and relative humidity (70%–95%).

### 2.2 Measurement of heading date, plant height, and leaf number

HD was the average number of days from sowing to the first panicle emergence per plant for each accession (n = 32). Average plant height, defined as the length from the soil surface to the panicle neck, was measured and recorded at maturity for each plant of each accession. Meanwhile, the average growth curve of leaf age was the number of mature leaves per plant per 5 days after seed germination for each accession in the phytotron.

### 2.3 The sequence analysis of flowering time–related genes

The FT-related genes in foxtail millet were identified on the basis of their homologs in Arabidopsis, rice, sorghum, and green foxtail. The queries of FT-related genes *AtCO* and *AtFT* in Arabidopsis as well as *OsGhd7*, *OsEhd1*, *OsHd1*, and *OsHd3a* in rice were used by reciprocal BLAST on the following databases: Arabidopsis (The Arabidopsis Information Resource, TAIR9), rice (Rice Genome Annotation Project, RGAP7), sorghum (Phytozome *Sorghum bicolor* v3.1.1), green foxtail (Phytozome *Setaria viridis* v1.1), and foxtail millet (Phytozome *Setaria italica* v2.2). A reciprocal BLASTP search was performed on two databases of Arabidopsis and rice to confirm the homologs, and then, a TBLASTN search queries were performed on sorghum, green foxtail, and foxtail millet genomes with an expectation value (E-value) threshold of 1e−20. Descriptions of putative homologous FT-related genes in the Arabidopsis, rice, sorghum, green foxtail, and foxtail millet datasets are summarized in **Supplementary Table 1**. The putative orthologs in foxtail millet were analyzed for amino acid similarity with rice flowering-related protein, and the nucleotide polymorphism analysis of these homologous genes between two foxtail millet genomes, Yugu1 (Doust et al., 2009; Bennetzen et al., 2012) and TT8 (Tsai et al., 2016), are also shown in **Supplementary Table 1**.

Leaves of 3-week-old seedling of nine accessions were collected for genomic DNA extraction using the cetyl-trimethyl ammonium bromide (CTAB) method with modification (Murray and Thompson, 1980). The DNA of each gene was amplified by DNA polymerase KOD Plus (TOYOBO Co., Ltd., Japan), purified by the MinElute system (Qiagen, Netherlands), and then sent for Sanger sequencing (MISSION BIOTECH Co., Ltd., Taipei, Taiwan). The deduced amino acid sequences of all putative genes were aligned by CLUSTALW and consequently subjected to construct a consensus neighbor-joining tree according to 1,000 times of bootstrapping by Molecular Evolutionary Genetics Analysis 7 (MEGA7) (Kumar et al., 2016). The gene structure also was drawn by using the Gene Structure Display Server 2.0 (Stothard, 2000).

### 2.4 Expression levels of flowering time–related genes by quantitative real-time PCR

To assess the expression levels of FT-related genes in different ATs under the SD condition, leaves were harvested on day 52 after seed germination at controlled temperatures of 30/25°C, 25/20°C, 20/15°C, and 15/13°C. To study the temporal expression of genes, leaves were collected starting on day 52 after seed germination at 5-day intervals until heading at controlled temperatures of 25/20°C and 20/15°C. Samples were collected 1 to 2 h after sunrise, before 8 a.m. The second leaf (−2 leaf) below the newly emerged mature leaf was collected for RNA extraction by using TRIzol reagent (Invitrogen, USA) and consequently Direct-zol RNA Miniprep (Zymo Research Co., USA). The first strand of complementary DNA was synthesized by using 2 µg of total RNA with the High-Capacity cDNA Reverse Transcription Kit (Applied Biosystems™, USA). Quantitative real-time PCR (qRT-PCR) was performed using a QuantiNova SYBR Green PCR kit (Qiagen, Netherlands) on the QuantStudio Real-Time PCR System (ThermoFisher). The sequences of gene-specific primers used for qRT-PCR are listed in **Supplementary Table 2**. The expression levels of FT-related genes were calculated by use of the 2^−ΔΔCT^ method (Livak and Schmittgen, 2001), and the foxtail millet *Sicullin* (Seita.3G037700) gene expression level was used for normalization (Martins et al., 2016).

### 2.5 Statistical analysis

HD, plant height, leaf number, and gene expression data were presented as the mean ± SD, and significant differences were analyzed following an LSD test or two-tailed Student’s *t*-test. A two-way ANOVA was used to estimate the influence of temperature, genotype, and temperature × genotype factor on the expression of each FT-related gene. Pearson’s correlation coefficient test was used to evaluate the correlation between the expression levels of FT-related genes and temperature factor in four accessions—Andhra Pradesh, Arie, TCS1, and TT8—as well as the correlation between the expression levels of FT-related genes in the four accessions. The coexpression gene regulatory network of FT-related gene expression levels upon changes in AT was constructed and visualized by correlation coefficient using Cytoscape (version 3.9.1).

## 3 Results

### 3.1 Variation in heading date is affected by genotypes and environments

**Figure 1:**
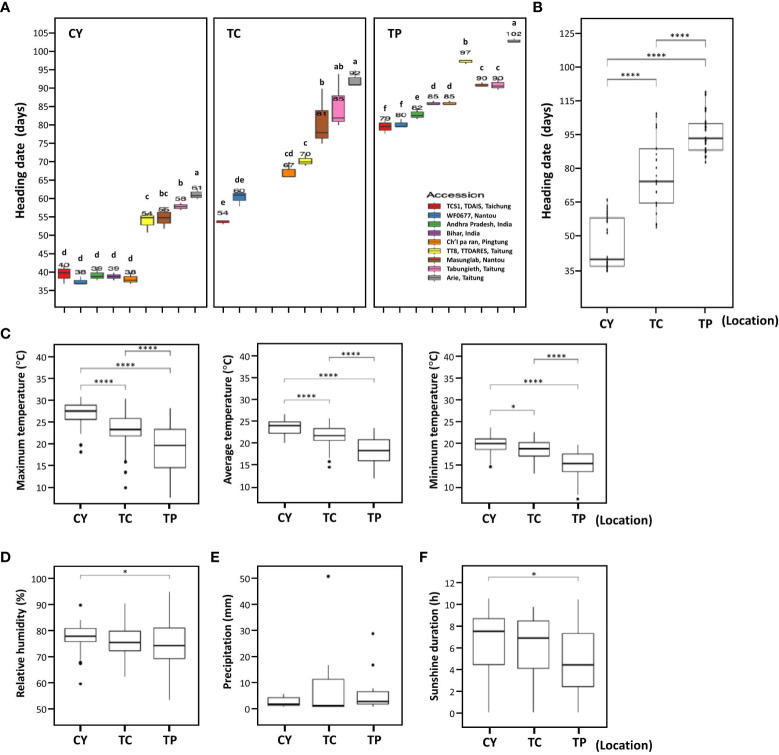
The heading dates of nine accessions grown at three environments. **(A)** The box plots of HDs for nine accessions grown at three locations. The numbers above each box plot represent the mean of HDs. CY indicates Chaiyi in south Taiwan, TC indicates Taichung in central Taiwan, and TP indicates Taipei in north Taiwan. In TC, accession Bihar and Andhra Pradesh were not evaluated. **(B)** The box plots of HDs for nine accessions at the three locations. **(C–F)** The meteorological data for three locations CY, TC, and TP. The maximum, average, and minimum temperature (°C) **(C)**, the relative humidity (%) **(D)**, precipitation (mm) **(E)**, and sunshine duration (h) **(F)** during cultivation period. The data were analyzed for significant differences using LSD test or two-tailed Student’s *t*-test. The different letters indicate statistical differences at P < 0.05. **** and * indicate significant differences at *P* < 0.0001 and *P* < 0.05, respectively.

To analyze the HDs of foxtail millet, nine accessions—five Taiwan landraces (Arie, Tabungieth, Ch’I pa ran, Masunglab, WF0677), two India landraces (Bihar and Andhra Pradesh), and two cultivars (TT8 and TCS1)—were selected on the basis of the previous study (Chen et al., 2017). These accessions were cultivated in CY in southern Taiwan, TC in central Taiwan, and TP in northern Taiwan from 2016 to 2020. In general, the HDs of these accessions were diverse with significant differences when cultivated in the same location, whether in CY or TP, although two Indica landraces were not evaluated in TC (**Figure 1A**). Among the nine accessions cultivated in CY, the earliest five accessions had HD ranging from 37.7 ± 1.20 days to 39.7 ± 2.50 days, whereas the latest HD accession was 61.3 ± 1.50 days (**Figure 1A**). In TC, the earliest HD accession was 53.7 ± 0.60 days, and the latest HD accession was 92.3 ± 2.30 days (**Figure 1A**). In TP, the earliest two accessions had HD ranging from 79.0 ± 2.00 days to 79.7 ± 1.20 days, whereas the latest HD accession was 102.3 ± 0.60 days (**Figure 1A**). Therefore, the genotypes of these nine accessions were associated with variation in HD.

The average HDs of the accessions in CY, TC, and TP were 46.9 ± 9.86 days, 72.9 ± 13.91 days, and 88.0 ± 7.68 days, respectively, indicating that the average HDs were significantly different among the three locations: It was earlier in CY in southern Taiwan than that in TC in central Taiwan and TP in northern Taiwan (**Figure 1B**). The HDs of these accessions also showed a similar trend in three different locations; therefore, HDs were correlated with latitude of locations. To understand what environmental cue affects HD, environmental factors including temperature, relative humidity, sunshine duration, and precipitation during foxtail millet growth period were analyzed. The maximum and minimum temperatures of CY were higher than those of TC and TP, and the average temperature among the three locations was significantly different (*P* < 0.0001, **Figure 1C**). The relative humidity and sunshine duration between CY and TP were also different (*P* < 0.05); however, other environmental factors were not significantly different between any two locations (**Figures 1D–F**). The different temperatures in the three locations suggested that AT might be the environmental cue causing HD variation in foxtail millet grown those three locations.

The earliest HD accessions were landrace WF0677 and cultivar TCS1. The HDs of these two accessions in CY, TC, and TP, from southern to northern Taiwan, ranged from 37.7 ± 1.20 days to 39.7 ± 2.50 days, 53.7 ± 0.60 days to 60.3 ± 2.10 days, and 79.0 ± 2.0 days to 79.7 ± 1.2 days, respectively (**Figure 1A**). The accession with the latest HD in CY, TC, and TP was Taiwan landrace Arie with an average HD of 61.3 ± 1.59 days, 92.3 ± 2.30 days, and 102.3 ± 0.6 days, respectively (**Figure 1A**). However, there are some exceptions, e.g., cultivar TCS1 in TC was earlier than landrace WF0677 but later than WF0677 in CY and TP. In addition, cultivar TT8 in CY and TC were earlier than landraces Masunglab and Tabungieth but later than Masunglab and Tabungieth in TP. Thus, some accessions are affected differently by environmental influences (G × E effects).

### 3.2 Ambient temperatures affecting plant growth and heading date

**Figure 2:**
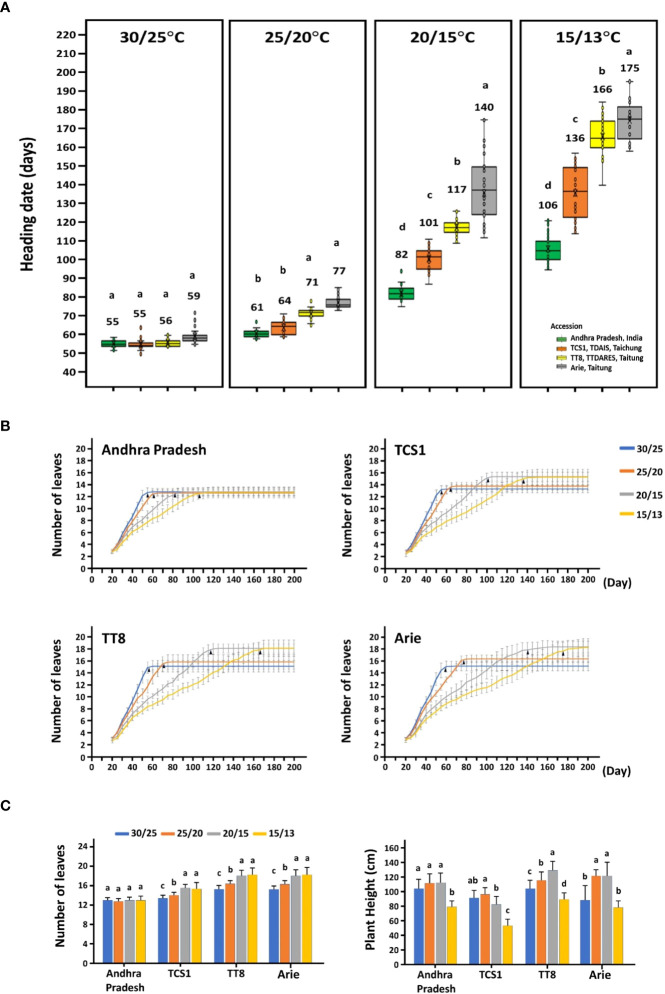
The heading date, leaf number, and plant height of four accessions grown under four different ambient temperature regimes in the phytotron. **(A)** The HDs of four accessions at four controlled day/night temperatures of 30/25°C, 25/20°C, 20/15°C, and 15/13°C. **(B)** The number of leaves were recorded from 20 to 200 days after germination for four accessions under four temperature regimes. The mean and standard deviation were estimated from 32 plants for each accession grown at each temperature. The black arrows indicated the average number of days of HDs at each temperature regime. **(C)** The leaf number and plant height of HD were recorded. The different letters indicate statistical differences at *P* < 0.05 by using the LSD test.

According to the HDs of foxtail millet planted in southern, central, and northern Taiwan, four accessions with early, moderate, and late HDs were selected: India landrace Andhra Pradesh (moderate early, 39 days in CY and 82 days in TP), Taiwan landrace Arie (very late, 61 days in CY, 92 days in TP, and 102 days in TP), and two cultivars TCS1 (extremely early, 40 days in CY, 54 days in TC, and 79 days in TP) and TT8 (moderate late, 54 days in CY, 70 days in TC, and 97 days in TP) (**Figure 1**). It was apparent that a high AT promoted HDs of the accessions to a range of 55.2 ± 1.80 days to 59.4 ± 4.05 days, whereas low AT delayed HDs to 106.1 ± 7.22 days to 174.7 ± 11.31 days (**Figure 2A**; **Supplementary Figure 1**). When these accessions were cultivated in the phytotron at 35/30°C, growth was hindered under this relatively high unfavorable AT condition and failed to heading (data not shown). In addition, the HD variation of the four accessions was also different under different AT, the HD variation was the largest when the AT was the lowest, and the HD variation was the smallest when the AT was the highest. The HD of the four accessions had little difference at 30/25°C, which was 4 days, and, at 25/20°C, it was 16 days, but, at 15/13°C, the HD difference was significant, which was 69 days. When the AT is higher than 30°C, it is not suitable for the growth of foxtail millet. The AT of 30/25°C is the optimum temperature for foxtail millet growth, and the other low ATs lead to differences in the HD of the four accessions.

The responses of these four accessions (genotypes) to various ATs were also different. The most obvious variation in HD under different ATs was Taiwan landrace Arie, whose HDs were 59.4 ± 4.05 days at 30/25°C and 174.7 ± 11.31 days at 15/13°C, with a difference of 115 days. In addition, the HDs of two cultivars, TCS1 and TT8, ranged from 54.9 ± 2.57 days to 56.1 ± 2.04 days at 30/25°C and 135.7 ± 13.66 days to 165.9 ± 9.59 days at 15/13°C, with a difference of 80 to 109 days. Furthermore, India landrace Andhra Pradesh had an HD difference of 51 days among the four ATs, which ranged from 55.2 ± 1.80 days to 106.1 ± 7.22 days (**Figure 2A**). The HDs of four accessions were significantly varied with various ATs. Thus, the variation of foxtail millet HD was affected by the interaction between environment temperature and genotype.

Heading, the transition from vegetative to reproductive stages, is associated with plant height in the *Poaceae* family and might be correlated with leaf number of the same genotype. The total leaf number and plant height were also affected by AT, but there were still differences among accessions (**Figures 2B, C**). The leaf number of the three accessions increased with the decrease of AT, only that of Andhra Pradesh remained relatively consistent (**Figure 2C**). The accession TT8 and Arie showed a trend of increasing plant height with decreasing AT from 30/25°C to 20/15°C, whereas Andhra Pradesh and TCS1 were the same from 30/25°C to 20/15°C but decreased in plant height at 15/13°C. Nevertheless, the plant heights of the four accessions all decreased significantly when the AT decreased to 15/13°C (**Figure 2C**). Therefore, leaf number and plant height affected by AT were genotype (accession) dependent.

### 3.3 Identification of genes associated with heading date in foxtail millet

A number of genes associated with FT have been identified that play key roles in the transition of vegetative and reproductive stages in Arabidopsis and rice (Cho et al., 2017; Shim and Jang, 2020; Cao et al., 2021), which were used to search for homologs in foxtail millet. A total of 2, 2, 3, and 3 OsGhd7 as well as of 6, 12, 9, and 10 OsEhd1 homologs were identified in rice, sorghum, green foxtail, and foxtail millet, respectively. A total of 6 AtCO/OsHd1 as well as of 6, 18, 19, 21, and 20 AtFT/OsHd3a homologs were identified in Arabidopsis, rice, sorghum, green foxtail, and foxtail millet, respectively. Thus, 10, 3, 6, and 20 homologs of *SiEhd1*, *SiGhd7*, *SiCO*, and *SiFT* were identified in foxtail millet compared with Arabidopsis, rice, sorghum, and green foxtail (**Supplementary Table 1**). Furthermore, foxtail millet homologs shared highly conserved amino acid similarities with rice FT-related proteins, such as OsGhd7 (47%–53%), OsEhd1 (24%–62%), OsHd1 (31%–76%), and OsHd3a (54%–87%), indicating that these homologs are well conserved in monocot species (**Supplementary Table 1**). However, comparison of nucleotide polymorphisms between Taiwan cultivar TT8 and Chinese cultivar Yugu1 showed that, in addition to *SiCO* gene family, *SiGhd7*, *SiEhd1*, and *SiFT* gene families were relatively divergent. The *SiGhd7*, *SiEhd1*, and *SiFT* gene family homologs have relatively more polymorphic sites, such as up to 31 sites in *SiGhd7-3*, 323 sites in *SiEhd1-2*, and 442 sites in *SiFT10.* By contrast, in the four *SiCO* gene family homologs, only *SiCO* and *SiCO4* have three and two polymorphic sites, respectively (**Supplementary Table 1**).

**Figure 3:**
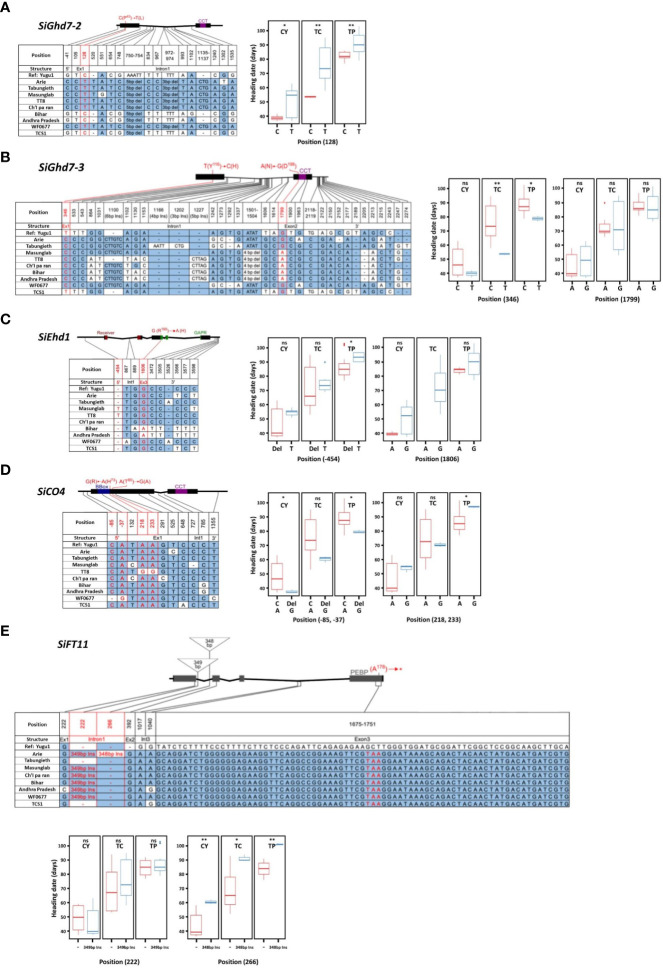
Association of allelic polymorphisms of flowering time-related genes with variation in heading date in foxtail millet. Allelic polymorphisms in *SiGhd7-2* **(A)**, *SiGhd7-3* **(B)**, *SiEhd1* **(C)**, *SiCO4* **(D)**, and *SiFT11* **(E)** in nine accessions was associated with HD at three locations. The nucleotide sequences were compared with those of reference accession Yugu1. Polymorphic nucleotides are indicated by different colors and labeled positions. Each gene scheme indicates its conserved functional domains and non-synonymous mutation sites. CY, TC, and TP indicate Chaiyi, Taichung, and Taipei, respectively. Data were analyzed for significant differences using a two-tailed Student’s *t*-test. **, *, and ns indicate significant difference at *P* < 0.01, *P* < 0.05, and no significant, respectively.

The associations between SNPs in FT-related genes and HD variations were analyzed by using nine accessions grown in southern (CY), central (TC), and northern (TP) Taiwan. Three homologs—*SiGhd7 (Seita.9G323600)*, *SiGhd7-2 (Seita.9G020100)*, and *SiGhd7-3 (Seita.7G007800)*—were identified in foxtail millet. According to the phylogenetic analysis of amino acid sequences, foxtail millet *SiGhd7* has high genetic similarity with green foxtail *SvGhd7 (Sevir.9G329600)*, sorghum *SbGhd7 (Sobic.001G298400)*, and rice *OsGhd7 (Os07g0261200)*. In addition, the gene structures of the three homologs were identified with two exons and one intron (**Supplementary Figures 2A, B**). One synonymous SNP on *SiGhd7*, one non-synonymous SNP on *SiGhd7-2*, and two non-synonymous SNPs on *SiGhd7-3* were identified in the nine accessions. The SNPs on the exon 1 of *SiGhd7-2* and *SiGhd7-3* were associated with HD variation (**Table 1**). A nucleotide substitution from C to T in exon 1 of *SiGhd7-2* in Taiwan landraces Arie, Tabungieth, Masunglab, Ch’I pa ran, WF0677, and cultivar TT8 caused a non-synonymous mutation P43L, which was highly associated with HD variation at three locations (CY, *P* < 0.05; TC and TP, *P* < 0.01) (**Figure 3A**, **Supplementary Figure 2C**). The nucleotide substitution from T to C in *SiGhd7-3* exon 1, resulting in a non-synonymous mutation from Tyr to His (Y116H), was associated with HD variation in two locations (TC, *P* < 0.01; TP, *P* < 0.05) (**Figure 3B**, **Supplementary Figure 2C**). Thus, two of the three Ghd7 homologs, the alleles of *SiGhd7-2* and *SiGhd7-3*, were associated with HD variation in these nine accessions.

**Table 1:** The association between single-nucleotide polymorphisms of flowering-related genes and heading date in foxtail millet.

Locus	Accession no.	Single-nucleotide polymorphism				Location		
		Position	Sites	Allele	Non-syn	CY	TC	TP
***SiGhd7***	Seita.9G323600	Exon 1	198	G→A	–	ns	ns	ns
***SiGhd7-2***	Seita.9G020100	Exon 1	128	C→T	P43L	*	**	**
***SiGhd7-3***	Seita.7G007800	Exon 1	346	T→C	Y116N	ns	**	*
		Exon 2	1,799	G→A	D198N	ns	ns	ns
***SiEhd1***	Seita.9G231000	5′UTR	−454	∇T	–	ns	ns	*
		Exon 3	1,806	G→A	R190H	ns	ns	ns
***SiCO***	Seita.4G122700	Exon 1	271	G→A	A91T	ns	ns	ns
***SiCO4***	Seita.1G228800	5′UTR	−85	ΔC	–	*	ns	*
		Exon 1	218	A→G	H73R	ns	ns	*
		Exon 1	233	A→G	T80A	ns	ns	*
***SiFT***	Seita.4G067600	Exon 1	12	∇6bp	4∇RD	ns	ns	ns
		Exon 1	168	C→G	D58E	ns	ns	ns
		Exon 2	392	G→A	V73I	ns	ns	ns
***SiFT11***	Seita.5G317600	Intron 1	222	∇349bp	–	ns	ns	ns
		Intron 1	266	∇348bp	–	**	*	**

∇ indicates insertion, and Δ indicates deletion. Non-syn indicates non-synonymous mutation.

** indicates P < 0.01, and * indicates P < 0.05. ns indicates not significant. The statistical differences were analyzed by using two-tailed Student’s t-test.

Location in CY for Chiayi, TC for Taichung, and TP for Taipei.

Ten *SiEhd1* homologs were identified in foxtail millet, among which *SiEhd1 (Seita.9G231000)* clustered with rice *OsEhd1 (Os10g463400)*, green foxtail *SvEhd1 (Sevir.9G230500)*, and sorghum *SbEhd1 (Sobic.001G227900)* with high genetic similarity. In addition, the Ehd1 homologs of these species all have the same gene structure of five exons and four introns, and their protein sequences all contain the conserved receiver and Golden2, *Arabidopsis RESPONSE REGULATOR* [ARR], and *Chlamydomonas* regulatory protein of P-starvation acclimatization response [Psr1] (GARP) domains (**Supplementary Figure 3**). Two SNPs were found in *SiEhd1* in the nine accessions; however, a single-nucleotide deletion in the 5’ untranslated region (5’UTR) was association with HD variation only when foxtail millet was grown in TP (**Table 1**, **Figure 3C**).

A total of six *SiCO* homologs were identified in foxtail millet. Among them, *SiCO (Seita.4G122700)* and *SiCO4 (Seita.1G228800)* contain two exons and one intron, which are highly similar to *AtCO* and *OsHd1*, and their amino acid sequences both contain B-box and CCT domains (**Supplementary Figure 4**). A non-synonymous mutation from Gly to Thr (A91T) in *SiCO* exon 1 in Indian landraces Bihar and Andhra Pradesh was not associated with HD variation (**Table 1**, **Supplementary Figure 4C**). Two SNPs, a 1-bp C deletion and a nucleotide substitution A to G, found in the 5′UTR of *SiCO4* of accession WF0677 were associated with HD variations in both CY (*P* < 0.05) and TP (*P* < 0.05) but no in TC (**Table 1**, **Figure 3D**). Another two SNPs on *SiCO4* exon 1 in accession TT8, leading to two non-synonymous mutations from His to Arg (H73R) and Thr to Ala (T80A), delayed HD in TP (*P* < 0.05) significantly but not the other two locations (**Table 1**, **Figure 3D**).

A total of 6, 18, 19, 21, and 20 of AtFT/OsHd3a homologs were identified in Arabidopsis, rice, sorghum, green foxtail, and foxtail millet, respectively. These 84 homologs could be divided into three subgroups: FLOWERING LOCUS T (FT)–like, TERMINAL FLOWER LIKE1 (TFL1)–like, and MOTHER OF FT and TFL1 (MFT)–like. Among 20 homologs in foxtail millet, 15 belonged to the FT-like subgroup and five belonged to the TFL1-like subgroup (**Supplementary Figure 5A**). *SiFT (Seita.4G067600)* and *SiFT11 (Seita.5G317600)* shared 87% and 58% amino acid similarity to rice *OsHd3a*, respectively, and were clustered in FT-like subgroup closely related to *AtFT* and *OsHd3a*. Moreover, the gene schemes of *SiFT* and *SiFT11* contain four exons and three introns, with a conserved phosphatidylethanolamine binding protein domain (**Supplementary Figures 5B, C**). Three SNPs in exon 1 and 2 of *SiFT* were identified in the nine accessions; however, none of them were associated with HD variation (**Table 1**). For *SiFT11*, all accessions had a 77-bp nucleotide difference in exon 3, resulting in an early stop codon, compared with the reference genome Yugu1 (**Figure 3E**). Two insertions were found in *SiFT11* intron 1; the first 349-bp insertion was not associated with HD, but the second 348-bp insertion was associated with HD variation at three locations (**Table 1**, **Figure 3E**).

### 3.4 Relationships between gene expression levels, ambient temperature, and heading date in foxtail millet

To clarify how AT affects the HD of foxtail millet, the expression levels of 14 FT-related genes were analyzed at four different controlled day/night temperatures on day 52 after seed germination, because foxtail millet entered the heading stage at 30/25°C. *SiGhd7*, *SiGhd7-2*, *SiEhd1*, *SiCO*, *SiCO4*, *SiFT*, and *SiFT11* are FT-related homologous genes, which were selected according to the previous study (**Table 1**). In addition, three temperature-regulated genes (*SiPHYB*, *SiPIF4*, and *SiPIF4-2*) and several genes involved in the circadian clock were included in the study. The expression levels of the 14 genes except for *SiPIF4* and *SiPIF4-2* were significantly affected by temperatures as shown by two-way ANOVA (**Table 2**). Except for *SiCO4*, the expression levels of the other 13 FT-related genes were genotype dependent. The expressions of all 14 tested genes were affected by the temperature × genotype interaction with significance (**Table 2**). In general, genotype, temperature, and genotype × temperature interactions explained the variation in the expression levels of these 14 FT-related genes (**Table 2**).

**Table 2:** Two-way ANOVA of temperature, genotype, and temperature × genotype factor in flowering time-related gene expression.

Gene	*Temperature* ^a^		*Genotype* ^b^		*Temperature* × *Genotype*	
	(F-value)		(F-value)		(F-value)	
***SiPHYB***	14.9822	****	16.8270	****	3.0965	**
***SiPRR1***	67.3390	****	13.9800	****	4.6252	***
***SiPRR59***	82.8025	****	34.6517	****	2.7757	*
***SiPRR73***	6.3498	**	45.8335	****	6.8334	****
***SiPRR95***	155.4840	****	18.4426	****	3.0614	**
***SiGhd7***	35.8128	****	15.4732	****	7.0149	****
***SiGhd7-2***	92.2262	****	55.6086	****	8.9129	****
***SiEhd1***	73.7460	****	21.2010	****	6.9200	****
***SiCO***	20.3000	****	26.4760	****	11.9910	****
***SiCO4***	10.7011	****	2.1264	ns	7.1338	****
***SiFT***	12.5145	****	12.1871	****	3.1934	**
***SiFT11***	17.4440	****	9.0437	****	9.3843	****
***SiPIF4***	1.7737	ns	30.3293	****	11.3527	****
***SiPIF4-2***	2.6968	ns	11.0645	****	6.7531	****

**^a^** indicates four ambient temperatures: 30/25°C, 25/20°C, 20/15°C, and 15/13°C (day/night).

**^b^** indicates four genotypes: accession Andhra Pradesh, TCS1, TT8, and Arie.

****, ***, **, and * indicate P < 0.0001, P < 0.001, P < 0.01, and P < 0.05, respectively. ns indicates not significant.

**Figure 4:**
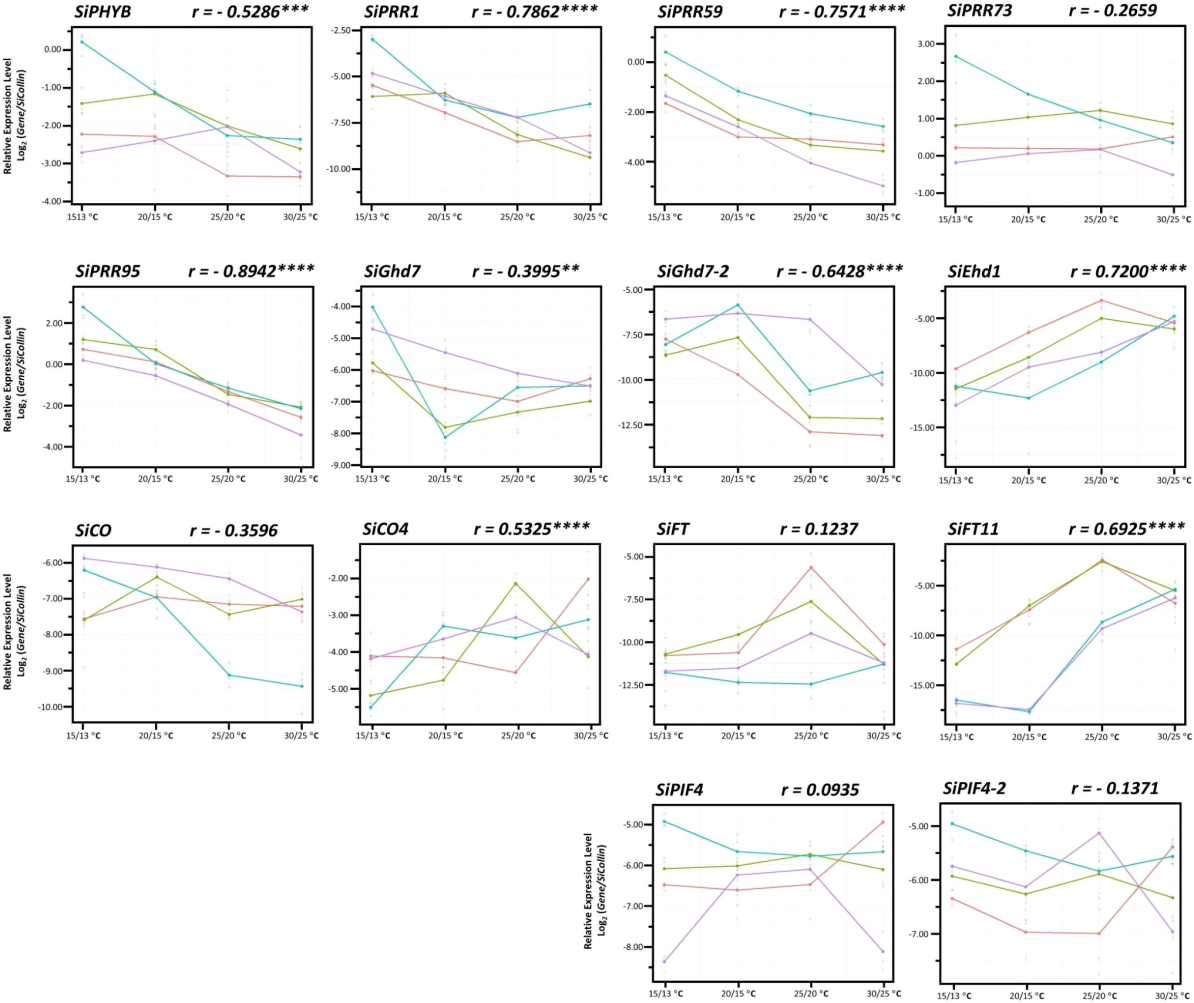
Expression of 14 flowering time-related genes in foxtail millet under four different ambient temperatures. The expression levels of 14 FT-related genes in four accessions—Andhra Pradesh (red), TCS1 (blue), TT8 (purple), and Arie (green)—from day 52 after seed germination under controlled day/night temperature of 30/25°C, 25/20°C, 20/15°C, and 15/13°C. The upper right corner of each gene expression level indicates correlation coefficient between all accessions and four ambient temperature changes. Data are presented as mean ± SD from three independent replicates. ****, ***, and ** indicate significant differences at *P* < 0.0001, *P* < 0.001, and *P* < 0.01 respectively.

Because the expression of these 14 FT-related genes was affected by AT, we analyzed whether the genes were negatively or positively regulated with AT. Nine of the 14 genes were highly correlated with changes in AT (**Figure 4**). The expression levels of six genes—*SiPRR95* (r = −0.8942, *P* < 0.0001), *SiPRR1* (r = −0.7862, *P* < 0.0001), *SiPRR59* (r = −0.7572, *P* < 0.0001), *SiGhd7-2* (r = −0.6428, *P* < 0.0001), *SiPHYB* (r = −0.5286, *P* < 0.001), and *SiGhd7* (r = −0.3995, *P* < 0.01)—were highly negatively correlated with changes in AT (**Table 3**, **Figure 4**). However, the expression levels of three genes—*SiEhd1* (r = 0.7200, *P* < 0.0001), *SiFT11* (r = 0.6925, *P* < 0.0001), and *SiCO4* (r = 0.5325, *P* < 0.0001)—were highly positively correlated with changes in AT (**Table 3**, **Figure 4**). In addition, the expression levels of some genes such as *SiFT*, *SiFT11*, and *SiEhd1* were significantly different at specific Ats, with the highest expression levels at 25/20°C and decreased expression levels at 30/25°C (**Figure 4**). Although these 14 genes are regulated by AT, the responses to different AT were varied.

**Table 3:** Association analyses of flowering time-related gene expression and environmental temperature in four foxtail millet accessions.

*Gene*	Ambient temperature									
	Andhra Pradesh		TCS1		TT8		Arie		All	
***SiPHYB***	−0.7773	**	−0.6693	*	−0.8637	***	−0.2159		−0.5286	***
***SiPRR1***	−0.8421	***	−0.8421	***	−0.6909	*	−0.9284	****	−0.7862	****
***SiPRR59***	−0.7125	**	−0.9069	****	−0.9500	****	−0.9500	****	−0.7571	****
***SiPRR73***	0.4318		0.2375		−0.9716	****	0.0000		−0.2659	
***SiPRR95***	−0.9500	****	−0.9500	****	−0.9716	****	−0.9716	****	−0.8942	****
***SiGhd7***	−0.2159		−0.3455		−0.2191		−0.8853	***	−0.3995	**
***SiGhd7-2***	−0.9069	****	−0.6693	*	−0.5830	*	−0.6262	*	−0.6428	****
***SiEhd1***	0.6478	*	0.8421	***	0.8205	**	0.9716	****	0.7200	****
***SiCO***	0.0432		0.2159		−0.8614	***	−0.9284	****	−0.3596	
***SiCO4***	0.4534		0.5830	*	0.6262	*	0.2159		0.5325	****
***SiFT***	0.3671		−0.1296		0.0216		0.4102		0.1237	
***SiFT11***	0.4966		0.7773	**	0.7773	**	0.8205	**	0.6925	****
***SiPIF4***	0.4966		0.0864		−0.6262	*	0.0864		0.0935	
***SiPIF4-2***	0.3671		−0.1080		−0.6693	*	−0.3455		−0.1371	

These gene expression levels and ambient temperature were analyzed by using correlation coefficient test, and significant differences were analyzed by using two-tailed Student’s t-test. ****, ***, **, and * indicate P < 0.0001, P < 0.001, P < 0.01, and P < 0.05, respectively.

The expression of FT-related genes in response to AT changes in the four accessions indicated genotype dependent (**Table 3**, **Figure 4**). Only six genes in the Indian landrace Andhra Pradesh but up to 12 genes in the Taiwan modern cultivar TT8 were significantly expressed by AT changes. Furthermore, the expression of these AT dependent genes was negatively correlated with AT (**Table 3**). The correlation between *SiGhd7* expression level and changes in AT (r = −0.8853, *P* < 0.001) was highly significant in Taiwan landrace Arie, whereas the correlation between *SiPHYB* expression level and changes in AT was significant in the other three accessions (r = −0.8637 to −0.6693). The expression levels of *SiFT11* correlated with AT in the three accessions (r = 0.8205 to 0.7773) but not in India landrace Andhra Pradesh. The expression of SiPRR37 influenced by AT was detected only in TCS1 but not in other accession. In TT8, the expression levels of two *SiCO* family members, *SiCO* (r = −0.8614, *P* < 0.001) and *SiCO4* (r = 0.6262, *P* < 0.05), were correlated with AT. In addition, the expression level of *SiPRR73* (r = −0.9716, *P* < 0.0001), *SiPIF4* (r = −0.6262, *P* < 0.05), and *SiPIF4-2* (r = −0.6693, *P* < 0.05) in TT8 was also correlated with changes in AT, which was unique to the other three accessions.

**Figure 5:**
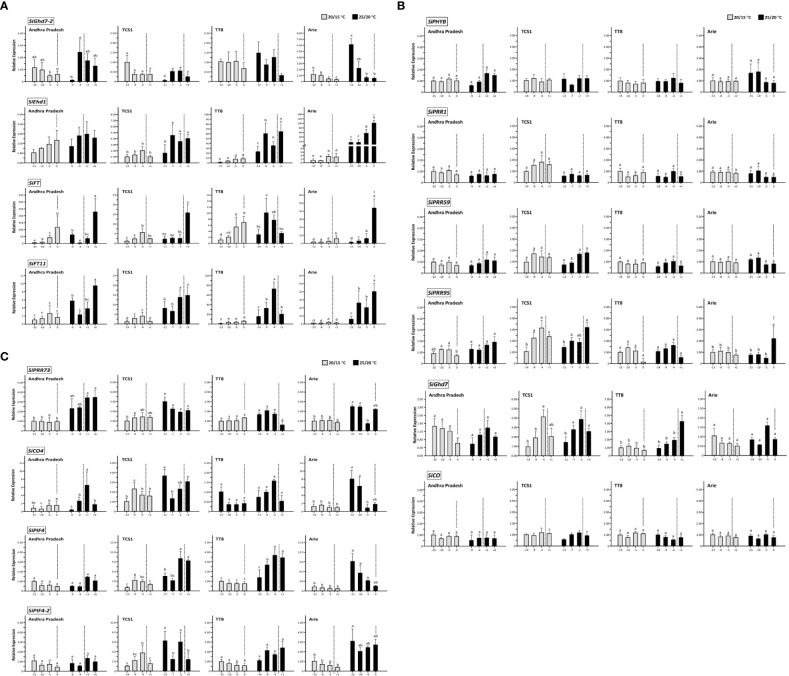
Temporal expression of 14 flowering time-related genes in foxtail millet heading stage. The expression levels of 14 FT-related genes in four accessions—Andhra Pradesh, TCS1, TT8, and Arie—at controlled day/night temperature of 20/15°C and 25/20°C around HD. **(A)** FT-related genes *SiGhd7-2*, *SiEhd1*, *SiFT*, and *SiFT11*. **(B)** FT-related genes *SiPHYB*, *SiPRR1*, *SiPRR59*, *SiPRR95*, *SiGhd7*, and *SiCO*. **(C)** FT-related genes *SiPRR73*, *SiCO4*, *SiPIF4*, and *SiPIF4-2*. The average HD of each accession was set to 0, and the dotted lines represent the average HD at each temperature regime; the negative and positive numbers represent days before and after HD, respectively. The expression levels of genes were normalized to the first time point of each accession at 20/15°C. Data are presented as mean ± SD from three independent replicates. The different letters indicate statistical differences at *P* < 0.05 by using LSD test.

The temporal expression of these 14 FT-related genes before and after HD was also analyzed with two temperature regimes of 20/15°C and 25/20°C. The temporal expression levels of four and six genes were significantly and non-significantly different, respectively (**Figures 5A,B**). The other four genes showed temporal differences in expression at 25/20°C but not at 20/15°C (**Figure 5C**). The expression levels of four genes—*SiGhd7-2*, *SiEhd1*, *SiFT*, and *SiFT11—*were highly correlated with HD. As HD approached, the expression of *SiGhd7-2* decreased, whereas the expression levels of *SiEhd1*, *SiFT*, and *SiFT11* increased, and the expression levels of these genes enhanced with the increased of AT, indicating that the changes in the expression of these genes were directly associated with HD (**Figure 5A**). Among the four accessions, the Taiwan landrace Arie had the largest HD variation under different ATs, and the expression levels of these four genes also had the most significant differences in Arie (**Figure 5A**). The expression levels of six genes—*SiPHYB*, *SiPRR1*, *SiPRR59*, *SiPRR95*, *SiGhd7*, and *SiCO—*in the four accessions had no significant changes before and after HD (**Figure 5B**). At the heading stage, high AT enhanced the expression of *SiPRR73*, *SiCO4*, *SiPIF4*, and *SiPIF4-2*, and the expression levels of the four accessions before and after HD were basically the same but significantly increased at 25/20°C compared with that at 20/15°C (**Figure 5C**).

### 3.5 Interaction of genes related to heading date in foxtail millet under environmental temperature change

**Figure 6:**
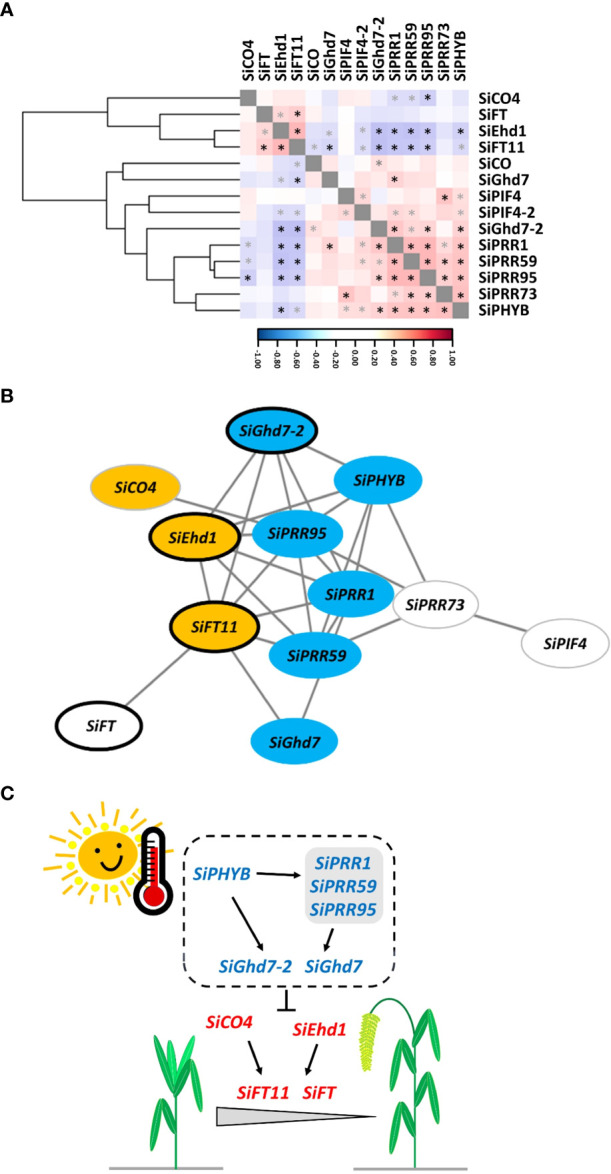
A coexpression gene regulatory network of flowering time-related genes in response to ambient temperature in foxtail millet. **(A)** Hierarchical clustering of 14 gene coexpression levels of four accessions—Andhra Pradesh, TCS1, TT8, and Arie—under changes in AT. The correlation coefficient scale color bar from blue to red indicates negative to positive correlation. Black and gray asterisks (*) indicate the statistical significance at *P* < 0.001 and *P* < 0.01, respectively, using Pearson’s correlation coefficient test. **(B)** The coexpression gene regulatory network was constructed with a Pearson’s correlation coefficient cutoff of *P* < 0.001 and visualized using Cytoscape v3.9.1. Each node represents a gene, and the edges represent the gene correlation. The node colors represent the significant positive correlation (orange), negative correlation (blue), and no significant correlation (white) between gene expression levels and AT, as shown in **Table 3**. The black frame of the node represents that the temporal expression of the gene is related to the HD and AT of foxtail millet, as shown in **Figure 5**. **(C)** Schematic model of AT affecting the HD in foxtail millet under SD conditions. When increasing AT, foxtail millet promotes HD through the regulation of genes. Genes labeled in blue and red represent gene expression levels negatively and positively correlated with AT, respectively.

The gene coexpression regulatory network was constructed to elucidate the effect of AT on the expression of FT-related genes and then on the HD of foxtail millet. The expression levels of these 14 genes at the four ATs were used to calculate the correlation coefficients of gene × gene interactions and analyzed by hierarchical clustering, which showed that these genes were divided into two groups (**Figure 6A**). The expression levels of *SiCO4*, *SiFT*, *SiEhd1*, and *SiFT11* were correlated each other in one group, and the expression levels of *SiCO*, *SiGhd7*, *SiPIF4*, *SiPIF4-2*, *SiGhd7-2*, *SiPRR1*, *SiPRR59*, *SiPRR95*, *SiPRR73*, and *SiPHYB* were correlated each other in the other group (**Figure 6A**). A transcriptional regulatory network consisting of 12 genes (nodes) and 27 interactions (edges) indicated that these genes involved in the regulation of foxtail millet HD were highly significantly correlated with AT by using Cytoscape v3.9.1 to set the correlation coefficient cutoff at *P* < 0.001 (**Figure 6B**). The nine genes [*SiCO4*, *SiEhd1*, and *SiFT11* (orange mark), which were significantly positively correlated with AT, and *SiGhd7-2*, *SiPHYB*, *SiPRR95*, *SiPRR1*, *SiPRR59*, and *SiGhd7* (blue mark), which were significantly negatively correlated with AT] were located at important positions in the network, as shown in **Table 3**. In addition, four genes—*SiGhd7-2*, *SiEhd1*, *SiFT11*, and *SiFT—*around the network were associated with HD (marked with black frame), as shown in **Figure 5**. Therefore, on the basis of previous studies and our results, a simplified model of the effect of AT changes on foxtail millet HD under SD conditions was established (**Figure 6C**). In this model, *SiPHYB* likely act as a temperature sensor together with *SiPRR* family members and promote the down-regulation of *SiGhd7-2* and *SiGhd7* when AT increases. Meanwhile, two positive regulators, *SiEhd1* and *SiCO4*, induced the expression of *SiFT11* and *SiFT*, thereby promoting HD (**Figure 6C**). The gene network established by combining the four accessions showed that these genes have certain degrees of correlation between different accessions, which provided a basis for the follow-up research on the FT-related genes of foxtail millet.

## 4 Discussion

In Taiwan, foxtail millet has been cultivated for more than 5,000 years. Because of the indigenous people preferences and the high adaptability of foxtail millet to various environments, the genetic diversity of landraces is very high, which is reflected in agronomic traits and grain quality (Chen et al., 2017; Kuo et al., 2018; Yin et al., 2019). HD variation among foxtail millet accessions were observed when cultivated in different locations under natural SD conditions in Taiwan (**Figure 1**). Although photoperiod and temperature were found to be important environmental factors regulating FT, other environmental factors interact to affect crop growth (Cho et al., 2017). Nine accessions grown in southern, central, and northern Taiwan exhibited significant differences in HD (**Figure 1**). The three environmental factors of relative humidity, precipitation, and sunshine duration had no significant difference among these characters. Although the relative humidity and sunshine duration were significantly different only between CY and TP (*P* < 0.05), the temperature was highly significantly different between the three environments in TC, CY, and TP (*P* < 0.001), suggesting that AT is the main factor affecting the HD of foxtail millet under SD conditions (**Figure 1**).

The four accessions grown at higher AT entered HD earlier, and the change in AT had different effects on HD of different accessions in the temperature-controlled phytotron (**Figure 2**). The India landrace Andhra Pradesh was the least affected by AT, and the average HD difference between 30/25°C and 15/13°C was 51 days. However, the other three accessions were significantly affected by AT with a difference of more than 80–115 days. Lower AT also obviously impeded plant growth, such as cultivar TT8 and landrace Arie delayed heading for 150 days at 15/13°C (**Figure 2**). Because the optimum temperature for foxtail millet cultivation is between 16°C and 25°C (Kumar et al., 2018). On the other hand, four accessions grew well at 30/25°C but failed at 35/30°C with growth arrest. Foxtail millet grows faster under higher ATs, and its growth rate has a linear relationship with the increase of AT in the optimum temperature range, which is similar to that of rice (Nagalla et al., 2021). FT is influenced by genetic variation that interacts with the environment, the so-called G × E interaction, where different varieties grow optimally in different environments (Sasaki et al., 2015). The results support the notion that HD variations of foxtail millet can be explained by different genotypes (gene, G), different locations (environment, E), and the interaction between genotypes and locations (G × E); especially, the environment factors have a significant effect on HD (**Figure 1**).

With the availability of reference genomes and the advent of genomic approaches of model plants such as Arabidopsis and rice, the genome information has allowed the comparison of flowering pathways in extending into other species (Doust et al., 2009; Bennetzen et al., 2012). Eight genes were selected to investigate their nucleotide polymorphism in nine accessions, including Taiwan and India accessions (**Table 1**). A nucleotide substitution from G to A in *SiCO* intron 1 results in the splicing variation that was widely distributed in landraces from European and Asia (Fukunaga et al., 2015), and the HD variation might result from the loss of function of the *SiCO* protein in foxtail millet (Liu et al., 2015). In this study, the allelic variation in these nine accessions at this nucleotide was not associated with HD at three locations in Taiwan. In contrast, the allelic polymorphisms of *SiGhd7-2*, *SiGhd7-3*, *SiEhd1*, *SiCO4*, and *SiFT11* were associated with HD at three locations in Taiwan (**Table 1**, **Figure 3**), indicating that these genes were candidates for FT-related genes in foxtail millet.

The expression levels of 14 tested genes in four accessions were significantly affected by temperature, genotype, and temperature × genotype interaction. Meanwhile, these FT-related genes expression, such as *SiGhd7-2*, *SiEhd1*, *SiFT*, and *SiFT11*, were also significantly associated with HD (**Table 2**, **Figures 4**, **5**). In Arabidopsis, thermal induction from 23°C to 27°C under SD conditions was sufficient to trigger flowering (Balasubramanian et al., 2006), and this trigger was accompanied by an increase in *AtFT* expression, suggesting that *AtFT* can be activated independent of the photoperiod pathway (Song et al., 2013). In rice, low temperature affects the expression of the floral pathway integrator gene *OsHd3a*. When the temperature was decreased from 27°C to 23°C, the *OsHd3a* mRNA expression was significantly reduced under both SD and LD conditions, indicating that the expression of *OsHd3a* is dependent on photoperiod and AT pathway (Song et al., 2012). In foxtail millet, change in the expression levels of *SiFT* and *SiFT11* associated with AT, thereby affecting HD under the SD condition (**Figures 4**, **5**).

The correlation of gene × gene interactions with AT results revealed that the expression levels of *SiPHYB* and *SiPRR* family members were significantly positively correlated with each other and with that of *SiGhd7* and *SiGhd7-2* and negatively correlated with that of *SiCO4* (**Figures 6A, B**). In addition, the expression levels of *SiGhd7* and *SiGhd7-2* were also significantly negatively correlated with the expression levels of *SiEhd1* and *SiFT11*, and the expression level of *SiEhd1* was significantly positively correlated with those of *SiFT11* and *SiFT* (**Figures 6A, B**). *AtPHYB* could sense temperature changes, and *OsPHYB* affected the expression of *OsGhd7* (Jung et al., 2016; Zheng et al., 2019; Nagalla et al., 2021). *OsGhd7* is temperature-regulated and mainly suppresses HD in rice because *OsGhd7* represses *OsEhd1* and *OsHd3a* (Xue et al., 2008; Zhao et al., 2015; Nagalla et al., 2021). Therefore, the expression of these FT-related genes such as *SiGhd7-2*, *SiEhd1*, and *SiFT11* in foxtail millet is similar to that in rice, and they are all important genes that regulate foxtail millet HD under high AT conditions (**Table 3**, **Figures 5**, **6**).

Foxtail millet has attracted attention in combating climate change to ensure food security because of its high adaptability to harsh environments compared with other crops. Establishing trait-associated gene regulatory pathways requires many functional validations to demonstrate the interaction between genes. However, studies on HD pathways in foxtail millet are still limited. In this study, variations in HD among nine accessions were observed when cultivated in southern, central, and northern Taiwan under the natural SD condition (**Figure 1**). Subsequently, the study demonstrated that under SD conditions in Taiwan, the HD of foxtail millet accession was significantly correlated with AT. The suitable AT for foxtail millet growth is between 13°C and 30°C, and a higher AT promotes earlier HD (**Figure 2**). The allelic polymorphisms in FT-related genes were also associated with HD variations (**Table 1**, **Figure 3**). Thus, this study explored the relationship between the expression levels of 14 key FT-related genes and AT and HD (**Table 2**, **Figures 4**, **5**). Through the correlation coefficients of the expression levels of each FT-related gene with AT, a coexpression regulatory network was identified that interacted and was affected by AT (**Table 3**, **Figure 6**). The study of coexpression regulatory network revealed key FT-related genes and their possible interactions in the regulation of foxtail millet HD under AT changes, which provided a preliminary understanding and molecular basis for elucidating the mechanism of foxtail millet HD under SD conditions (**Figure 6**). This study provides a new understanding of possible flowering pathways in foxtail millet and is advisable to apply candidate gene approaches for successful breeding strategies for shift production to increase yield in response to global warming.

## Data availability statement

The raw data supporting the conclusions of this article will be made available by the authors, without undue reservation.

## Author contributions

Y-CH, Y-tW, and Y-cC performed the experiments and analyzed the data. Y-rC collected the plant materials. Y-rL designed the research. Y-CH wrote the manuscript, and Y-CH, H-yH, T-FH, and Y-rL co-revised the manuscript. All authors discussed the results and commented on the manuscript. All authors contributed to the article and approved the submitted version.
