# Supplementary material for: How ambient temperature affects the heading date of foxtail millet (Setaria italica)
Source: Front Plant Sci. 2023 Mar 2;14:1147756. doi: 10.3389/fpls.2023.1147756 (PMC10018198; doi:10.3389/fpls.2023.1147756)
Supplement: Supplementary file 1 [file DataSheet_1.pdf]

SUPPLEMENTARY MATERIAL

TITLE:

How Ambient Temperature Affects the Heading Date of Foxtail Millet (*Setaria italica*)

SUPPLEMENTARY FIGURES AND TABLES

Supplemental Table 1. Homologs of flowering time genes in *Arabidopsis*, rice, sorghum, green foxtail, and foxtail millet.

Supplemental Table 2. Gene name, accession number, and primer pair used for real-time PCR.

Supplemental Figure 1. Phenotypes of foxtail millet on the 61st day after seed germination at four different ambient temperatures.

Supplemental Figure 2. Genetic analysis of the *Ghd7* homologs.

Supplemental Figure 3. Genetic analysis of the *Ehd1* homologs

Supplemental Figure 4. Genetic analysis of the *CO* homologs.

Supplemental Figure 5. Genetic analysis of the *FT* homologs.

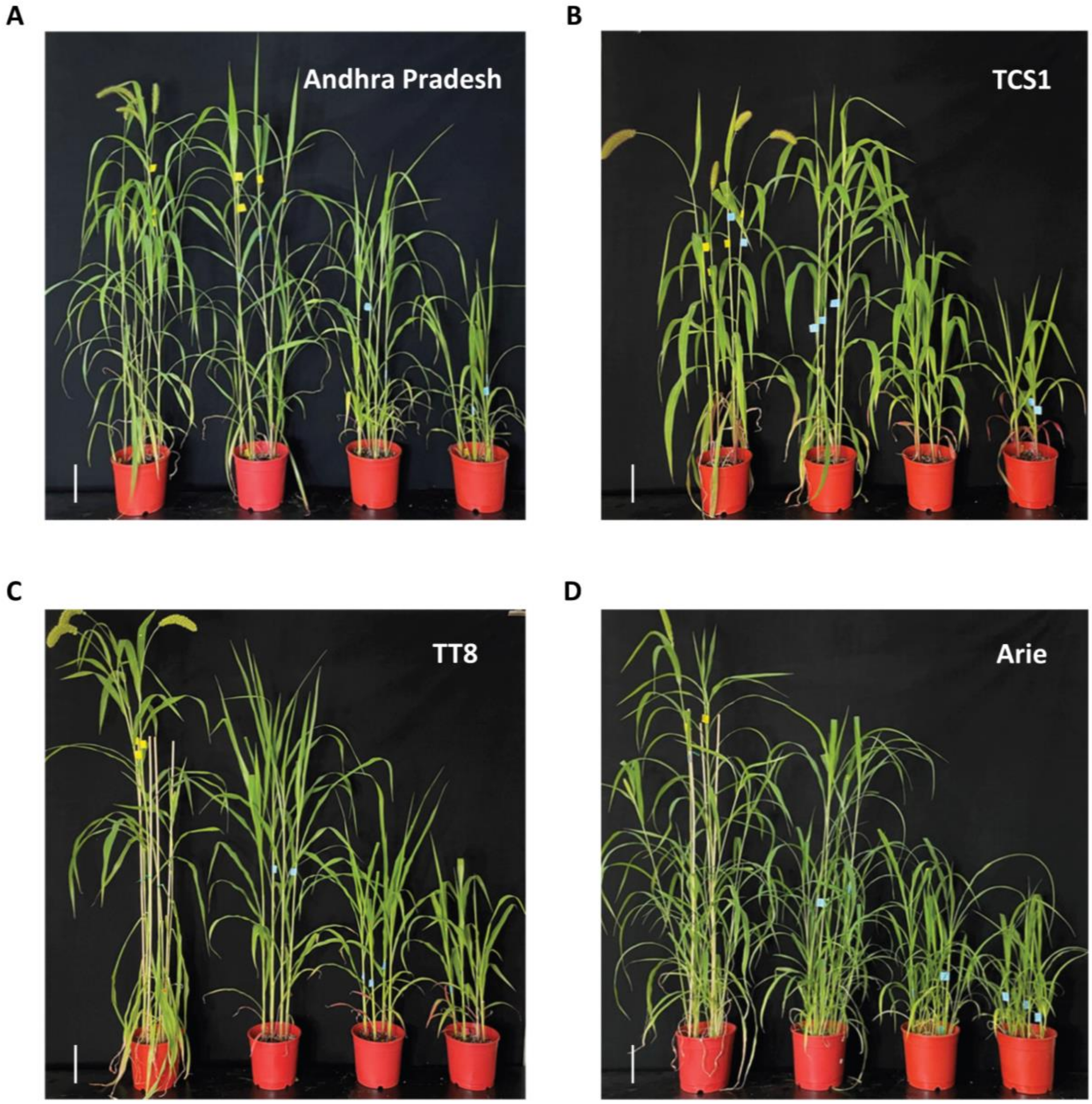

Supplementary Figure 1. Phenotypes of foxtail millet on the 61st day after seed germination at four different ambient temperatures. The four accessions are Andhra Pradesh **(A)**, TCS **(B)**, TT8 **(C)** and Arie **(D)** phenotypes of plants grown at different ATs in the phytotron (n=32). These plants were photographed on the 61st day after foxtail millet sowing. From left to right are plants grown at 30/25, 25/20, 20/15 and 15/13℃ for each accession. The bottom left is the scale bar representing 10 cm.

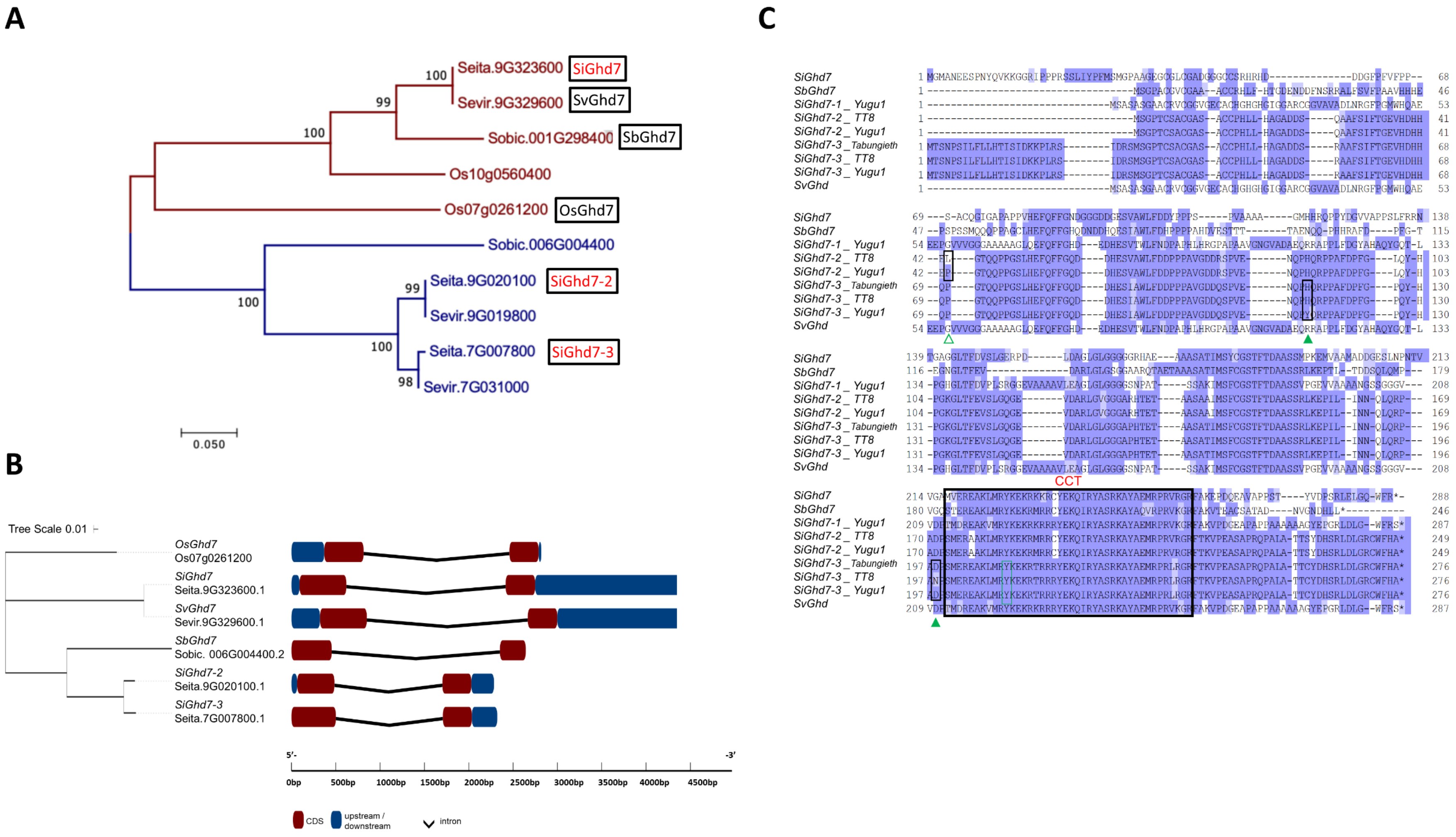

Supplementary Figure 2. Genetic analysis of the *Ghd7* homologs.

(A) The phylogenetic tree of the *Ghd7* homologs constructed by neighbor-joining. **(B)** The genetic relationships and gene structure of the *Ghd7* homologs. Blue box, red box, and black line indicate UTR, exon, and intron, respectively. **(C)** Sequence alignment of predicted amino acids of the *Ghd7* homologs. The black box represents the conserved amino acids of CCT (*CONSTANS*, *CO*-like, and *TOC1*) domain in the *Ghd7* homologs. The open triangle indicates the amino acid substitution (L→P) in *SiGhd7-2* between cultivar TT8 and Yugu1. The solid green triangles indicate the amino acid substitutions (H→Y) and (D→N) in *SiGhd7-3* between Tabungieth, TT8, and Yugu1. The prefixes of the locus names represent as following, *Os* for *Oryza sativa*, *Sb* for *Sorghum bicolor*, *Si* for *Setaria italica*, and *Sv* for *Setaria viridis.*

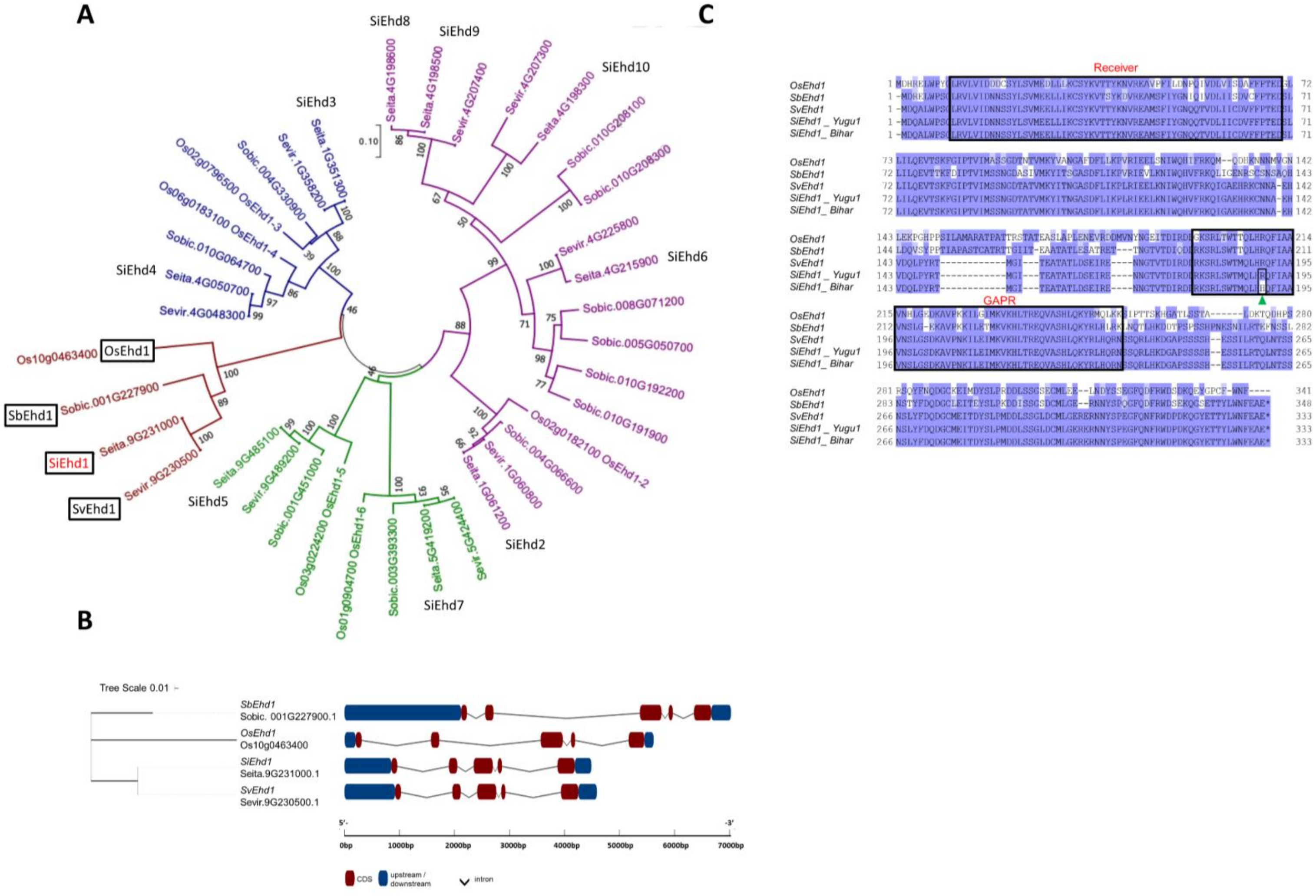

Supplementary Figure 3. Genetic analysis of the *Ehd1* homologs.

(A) The phylogenetic tree of the *Ehd1* homologs constructed by neighbor-joining. **(B)** The genetic relationships and gene structure of the *Ehd1* homologs. Blue box, red box, and black line indicate UTR, exon, and intron, respectively. **(C)** Sequence alignment of predicted amino acids of the *Ehd1* homologs. Two black boxes represent the conserved amino acids of Receiver and GAPR (*Golden2, Arabidopsis RESPONSE REGULATOR [ARR]*) domain in the *Ehd1* homologs, respectively. The solid green triangle indicates the amino acid substitution (R→H) in *SiEhd1* between Yugu1 and Bihar. The prefixes of the locus names represent as following, *Os* for *Oryza sativa*, *Sb* for *Sorghum bicolor*, *Si* for *Setaria italica*, and *Sv* for *Setaria viridis*.

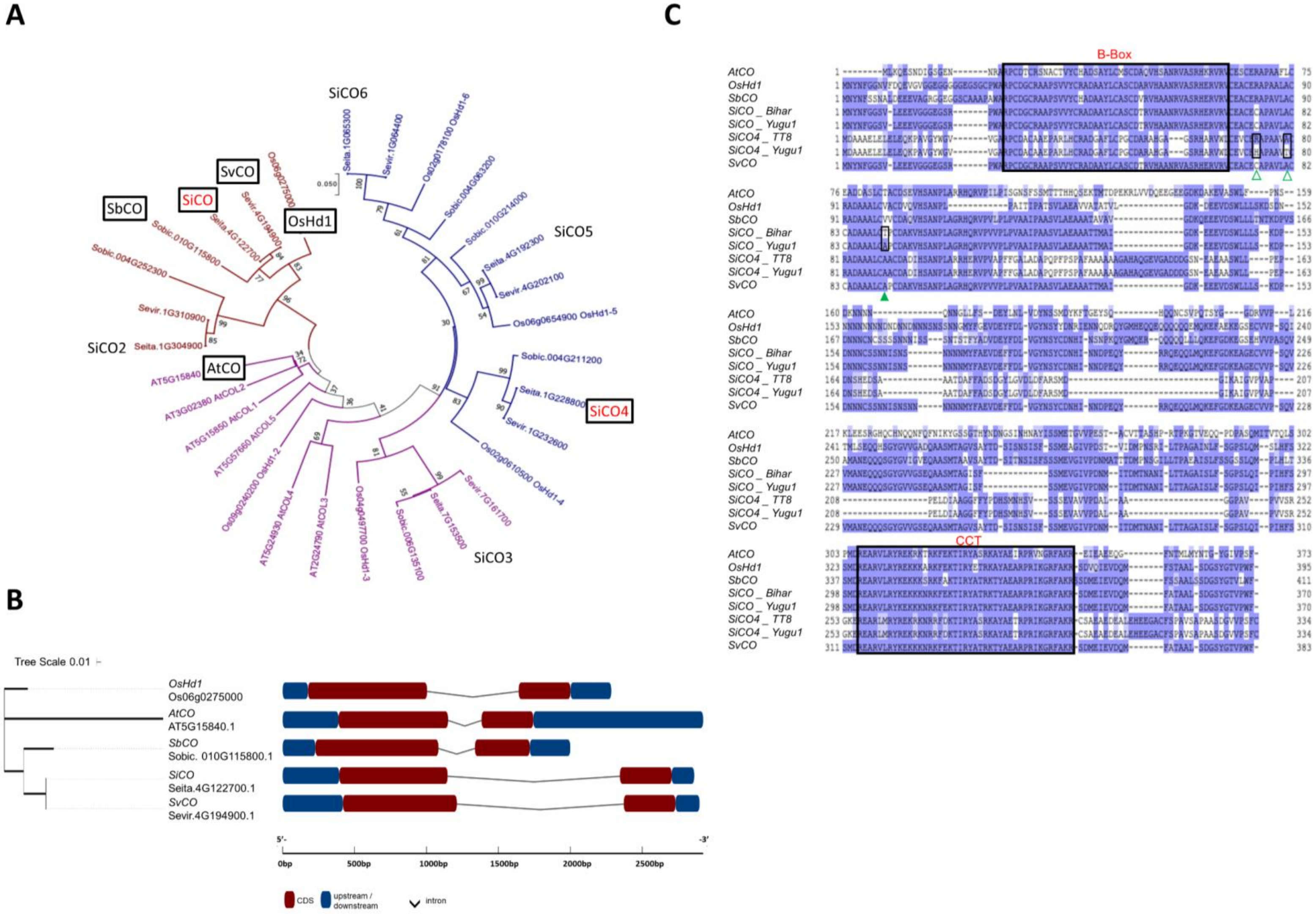

Supplementary Figure 4. Genetic analysis of the *CO* homologs.

(A) The phylogenetic tree of the *CO* homologs constructed by neighbor-joining. **(B)** The genetic relationships and gene structure of the *CO* homologs. Blue box, red box, and black line indicate UTR, exon, and intron, respectively. **(C)** Sequence alignment of predicted amino acids of the *CO* homologs. Two black boxes represent the conserved amino acids of B-box and CCT (*CONSTANS, CO-like*, and *TOC1*) domain in the *CO* homologs, respectively. The green triangle indicates the amino acid substitution (T→A) in *SiCO* between Bihar and Yugu1. Two open triangles indicate the amino acid substitutions (R→H) and (A→T) in *SiCO4* between two cultivars TT8 and Yugu1. The prefixes of the locus names represent as following, *Os* for *Oryza sativa*, *Sb* for *Sorghum bicolor*, *Si* for *Setaria italica*, and *Sv* for *Setaria viridis*.

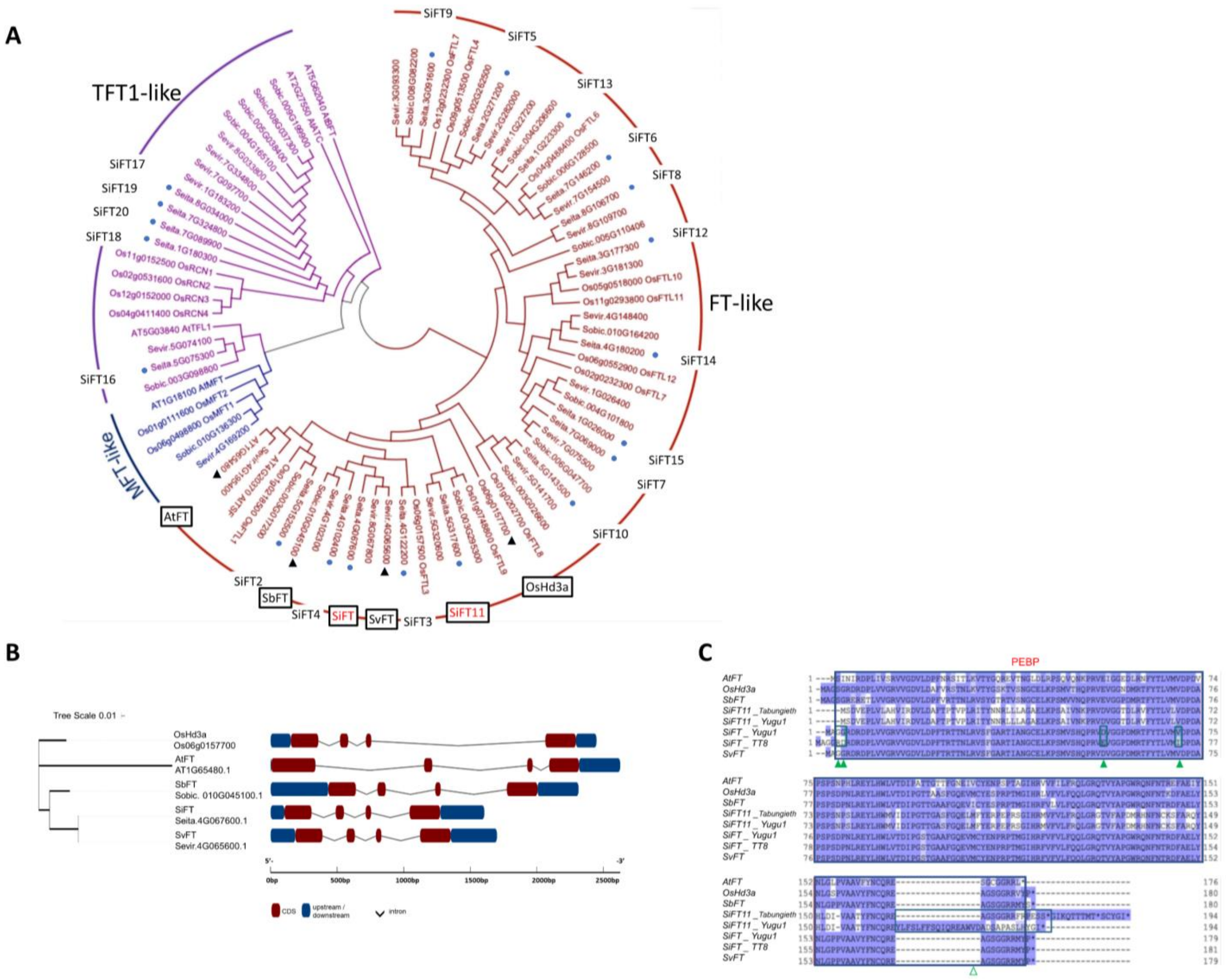

Supplementary Figure 5. Genetic analysis of the *FT* homologs.

(A) The phylogenetic tree of the *FT* homologs constructed by neighbor-joining. These homologs can be divided into three groups, FLOWERING LOCUS T (FT)-like, TERMINAL FLOWER LIKE1 (TFL1)-like, and MOTHER OF FT and TFL1 (MFT)-like. **(B)** The genetic relationships and genes structure of the *FT* homologs. Blue box, red box, and black line indicate UTR, exon, and intron, respectively. **(C)** Sequence alignment of predicted amino acids of the *FT* homologs. The black box represents conserved amino acids of PEBP (phosphatidylethanolamine binding protein) domain in the *FT* homologs. Four solid green triangles indicate the insertion of two amino acids RD, two amino acid substitutions (D→E), and (V→I) in *SiFT* between Yugu1 and TT8. The open triangle indicates a fragment of amino acid substitutions and early stop codons in *SiFT11* between Tabungieth and Yugu1. The prefixes of the locus names represent as following, *Os* for *Oryza sativa*, *Sb* for *Sorghum bicolor*, *Si* for *Setaria italica*, and *Sv* for *Setaria viridi.*

Supplementary Table 1. Homologs of flowering time genes in *Arabidopsis*, rice, sorghum, green foxtail, and foxtail millet.

Arabidopsis		Rice		Sorghum		Green Foxtail		Foxtail Millet					
Columbia		Nipponbare		BTX623		A10		Yugu1					
Locus	*Accession No.* [a]	*Locus*	*Accession No.* [a]	*Locus*	*Accession No.* [a]	*Locus*	*Accession No.* [a]	*Locus*	*Accession No.* [a]	Genomic Sequence length (bp)	Protein Length (aa) [b]	amino acid identity vs Os (%) [c]	No. of Polymorphic nucleotide in TT8 [d]
		OsGhd7	Os07g0261200	***SbGhd7***	Sobic.006G004400	***SvGhd7***	Sevir.9G329600	***SiGhd7***	Seita.9G323600	4801	286	53	2
		OsGhd7-2	Os10g0560400	***SbGhd7-2***	Sobic.001G298400	***SvGhd7-2***	Sevir.9G019800	***SiGhd7-2***	Seita.9G020100	2285	248	53	22
						SvGhd7-3	Sevir.7G031000	***SiGhd7-3***	Seita.7G007800	2322	176	47	31
		OsEhd1	Os10g0463400	***SbEhd1***	Sobic.001G227900	***SvEhd1***	Sevir.9G230500	***SiEhd1***	Seita.9G231000	4490	332	62	1
		OsEhd1-2	Os02g0182100	***SbEhd1-2***	Sobic.004G066600	***SvEhd1-2***	Sevir.1G060800	***SiEhd1-2***	Seita.1G061200	4772	634	33	323
		OsEhd1-3	Os02g0796500	***SbEhd1-3***	Sobic.004G330900	***SvEhd1-3***	Sevir.1G358200	***SiEhd1-3***	Seita.1G351300	4514	679	33	0
		OsEhd1-4	Os06g0183100	***SbEhd1-4***	Sobic.010G064700	***SvEhd1-4***	Sevir.4G048300	***SiEhd1-4***	Seita.4G050700	4429	666	34	0
		OsEhd1-5	Os03g0224200	***SbEhd1-5***	Sobic.001G451000	***SvEhd1-5***	Sevir.9G489200	***SiEhd1-5***	Seita.9G485100	6530	687	31	69
		OsEhd1-6	Os01g0904700	***SbEhd1-6***	Sobic.003G393300	***SvEhd1-6***	Sevir.5G424400	***SiEhd1-6***	Seita.4G215900	5307	701	31	71
				SbEhd1-7	Sobic.008G071200	***SvEhd1-7***	Sevir.4G225800	***SiEhd1-7***	Seita.5G419200	5551	578	31	121
				SbEhd1-8	Sobic.005G050700	***SvEhd1-8***	Sevir.4G207400	***SiEhd1-8***	Seita.4G198600	5099	696	30	62
				SbEhd1-9	Sobic.010G192200	***SvEhd1-9***	Sevir.4G207300	***SiEhd1-9***	Seita.4G198500	5094	696	31	301
				SbEhd1-10	Sobic.010G208100			***SiEhd1-10***	Seita.4G198300	6487	540	24	1
				SbEhd1-11	Sobic.010G191900								
				SbEhd1-12	Sobic.010G208300								
AtCO	AT5G15840	***OsHd1***	Os06g0275000	***SbCO***	Sobic.010G115800	***SvCO***	Sevir.4G194900	***SiCO***	Seita.4G122700	2862	370	76	3
AtCOL1	AT5G15850	***OsHd1-2***	Os09g0240200	***SbCO2***	Sobic.004G252300	***SvCO2***	Sevir.1G310900	***SiCO2***	Seita.1G304900	5527	385	52	0
AtCOL2	AT3G02380	***OsHd1-3***	Os04g0497700	***SbCO3***	Sobic.006G135100	***SvCO3***	Sevir.7G161700	***SiCO3***	Seita.7G153500	1944	326	39	0
AtCOL3	AT2G24790	***OsHd1-4***	Os02g0610500	***SbCO4***	Sobic.004G211200	***SvCO4***	Sevir.1G232600	***SiCO4***	Seita.1G228800	1593	334	38	2

AtCOL4	AT5G24930	***OsHd1-5***	Os06g0654900	***SbCO5***	Sobic.010G214000	***SvCO5***	Sevir.4G202100	***SiCO5***	Seita.4G192300	1833	372	31	0
AtCOL5	AT5G57600	***OsHd1-6***	Os02g0178100	***SbCO6***	Sobic.004G063200	***SvCO6***	Sevir.1G064400	***SiCO6***	Seita.1G065300	1969	386	32	0
AtFT	AT1G65480	***OsFTL1***	Os01g0218500	***SbFT***	Sobic.010G045100	***SvFT***	Sevir.4G065600	**SiFT**	Seita.4G067600	1606	178	87	12
TSF	AT4G20370	***OsHd3a***	Os06g0157700	***SbFT2***	Sobic.003G017200	***SvFT2***	Sevir.4G195400	***SiFT2***	Seita.5G152500	7476	173	80	120
MFT	AT1G18100	***OsFTL3***	Os06g0157500	***SbFT3***	Sobic.006G047700	***SvFT3***	Sevir.4G102300	***SiFT3***	Seita.4G122200	1493	177	81	1
ATC	AT2G27550	***OsFTL4***	Os09g0513500	***SbFT4***	Sobic.002G262500	***SvFT4***	Sevir.2G282000	***SiFT4***	Seita.4G102400	1845	188	71	0
TFL1	AT5G03840	***OsFTL6***	Os04g0488400	***SbFT5***	Sobic.004G206600	***SvFT5***	Sevir.7G154500	***SiFT5***	Seita.2G271200	7060	178	60	2
BFT	AT5G62040	***OsFTL7***	Os12g0232300	***SbFT6***	Sobic.006G128500	***SvFT6***	Sevir.1G227200	***SiFT6***	Seita.7G146200	2652	174	68	12
		OsFTL8	Os01g0202700	***SbFT7***	Sobic.008G082200	***SvFT7***	Sevir.7G075500	***SiFT7***	Seita.7G069000	2778	184	54	136
		OsFTL9	Os01g0748800	***SbFT8***	Sobic.003G026600	***SvFT8***	Sevir.8G109700	***SiFT8***	Seita.8G106700	2311	177	66	9
		OsFTL10	Os05g0518000	***SbFT9***	Sobic.003G295300	***SvFT9***	Sevir.3G093300	***SiFT9***	Seita.3G091600	1257	177	60	52
		OsFTL11	Os11g0293800	***SbFT10***	Sobic.009G199990	***SvFT10***	Sevir.5G141700	***SiFT10***	Seita.5G143500	8850	220	58	442
		OsFTL12	Os06g0552900	***SbFT11***	Sobic.005G110406	***SvFT11***	Sevir.5G320600	***SiFT11***	Seita.5G317600	1746	179	58	149
		OsFTL13	Os02g0232300	***SbFT12***	Sobic.010G164200	***SvFT12***	Sevir.3G181300	***SiFT12***	Seita.3G177300	1708	167	61	0
		OsMFT1	Os06g0498800	***SbFT13***	Sobic.004G101800	***SvFT13***	Sevir.8G067800	***SiFT13***	Seita.1G223300	2268	174	66	56
		OsMFT2	Os01g0111600	***SbFT14***	Sobic.010G136300	***SvFT14***	Sevir.4G148400	***SiFT14***	Seita.4G180200	2606	173	60	27
		OsRCN1	Os11g0152500	***SbFT15***	Sobic.003G098800	***SvFT15***	Sevir.1G026400	***SiFT15***	Seita.1G026000	1601	184	58	1
		OsRCN2	Os02g0531600	***SbFT16***	Sobic.005G038400	***SvFT16***	Sevir.4G169200	***SiFT16***	Seita.5G075300	2147	171	55	0
		OsRCN3	Os12g0152000	***SbFT17***	Sobic.004G165100	***SvFT17***	Sevir.5G074100	***SiFT17***	Seita.8G034000	1470	173	61	1
		OsRCN4	Os04g0411400	***SbFT18***	Sobic.008G037300	***SvFT18***	Sevir.8G033800	***SiFT18***	Seita.1G180300	1728	173	58	1
				SbFT19	Sobic.006G068300	***SvFT19***	Sevir.1G183200	***SiFT19***	Seita.7G324800	1688	173	61	110
						SvFT20	Sevir.7G334800	***SiFT20***	Seita.7G089900	1743	173	57	7
						SvFT21	Sevir.7G097700						

[a] The following databases are used for BLAST searches: *Arabidopsis* (The Arabidopsis Information Resource, TAIR9), Rice (Rice Genome Annotation Project, RGAP7), Sorghum (Phytozome *Sorghum bicolor* v3.1.1), green foxtail (Phytozome *Setaria viridis* v1.1), and foxtail millet (Phytozome *Setaria*

italica v2.2). [b] The homologous protein length is in amino acid number. [c] Amino acid similarity between foxtail millet family genes and representative proteins of rice (Os). [d] Number of nucleotide polymorphism differences between two foxtail millet accessions, TT8 and Yugu1.

Supplementary Table 2. Locus name, accession number, and primer pair used for real-time PCR.

Gene name	acession number	Forward Primer Sequence (5'→3')	Reverae Primer Sequence (5'→3')
SiPHYB	Seita.9g427800	TTCAGGTTTGCCTGTCCTGG	ATGTGCTTAGCCCAATGCCT
SiPRR1	Seita.1g236100	TGAGGAAACATGCAGTCGCT	CTCGTACCCTTGGCCTTGTT
SiPRR37	Seita.2g444300	GGGACAGGATTTGTGGGGAG	ACTGCCATTGGAGCCTTGTT
SiPRR73	Seita.9g445200	GCGGAAAGATCGGAACTTCG	AGATTGTCGCACAAACTGCC
SiPRR59	Seita.8g040100	CCGTGAAGCTGCCTTGATGA	ACTCTCGGTCTTTGCTCTGC
SiPRR95	Seita.2g286100	GTTCCTGAAGCCCCAAATGC	GCTCCGCGAGTAATTTCCTG
SiGhd7	Seita.9g323600	GATTCGCCAAGGTGCCTGA	TATGAACGGAACCACCCGAG
SiGhd7-2	Seita.9g020100	GCCTATGCAGAGATGAGACCC	GATCATAGCTGGTCGTTGCCA
SiEhd1	Seita.9g231000	ATGCTGGGAGAACGAGAACG	TTCCGCCTCGAAATTCCACA
SiCO	Seita.4g122700	AGCACGACCAAGGATCAAGG	TGGGACAGTACCATAGCTACCA
SiCO4	Seita.1g228800	CTTCTGCTGAGGGAGAGACG	CGCATGCGTTCAGATCCATT
SiFT	Seita.4g067600	GGCGCCAGAACTTCAACACC	TAGGGGTACATCCTCCTGCCG
SiFT11	Seita.5g317600	GGTGTTTGTGCTGTTCCGAC	GGCCACAATGTCTAGGTGGT
SiPIF4	Seita.3g384900	GTCCATGCCTAGGATGACGTT	GTAATGCATGGTGGAAGGCG
SiPIF4-2	Seita.9g160700	AGAGCCAGAACAACCGGAAG	ATGTCCTTGTCGAGCGAGTC
SiCullin	Seita.3g037700	TATGGGTCATCAACAGCTTGTC	GTAGTCCCTCGTGATGAGATCC
